# Using Quantitative Polymerase Chain Reaction (qPCR) to Identify a Myriad of Carbapenemase Genes in Fresh Cow Dung in Bangladesh

**DOI:** 10.7759/cureus.54644

**Published:** 2024-02-21

**Authors:** Mamun Al Asad, Ayasha Siddique Shanta, Kakoli Akter, Marnusa Binte Habib, Shamsun Nahar, Mainul Haque, Santosh Kumar, Salequl Islam

**Affiliations:** 1 Microbiology, Jahangirnagar University, Dhaka, BGD; 2 Dentistry, Karnavati University, Karnavati Scientific Research Center, Gandhinagar, IND; 3 Pharmacology and Therapeutics, National Defence University of Malaysia, Kuala Lumpur, MYS; 4 Periodontology and Implantology, Karnavati University, Karnavati School of Dentistry, Gandhinagar, IND; 5 Optometry and Vision Science, University of New South Wales, Faculty of Medicine and Health, Sydney, AUS

**Keywords:** carbapenemase-resistant genes, bangladesh, fresh, polymerase chain reaction, measurable, cow dung, cattle farm, qpcr, mbl, args

## Abstract

Introduction

The emergence of antimicrobial resistance (AMR) is driven by the selection pressure of frequent uses of antimicrobial agents in healthcare, the food chain, agriculture, fishery, and the food animal industry, which poses a serious health risk for transmission-linked humans and the surrounding environment. Livestock, particularly cattle, play an essential role in the food sector in Bangladesh. The food-animal chains can be the potential routes of exposure to AMR-microorganisms for every domain of one health. Antimicrobial resistance genes (ARGs) can impart a reservoir of AMR within the food supply chain, even without pathogenic microorganisms. This study investigated the history of infection for the last six-month period of antimicrobials utilized in cattle farms and the distribution of selected carbapenemase resistance genes, namely, *bla-KPC*, *bla-IMP*, *bla-VIM*, *bla-NDM-1*, *bla-SIM*, *bla-GIM*, *bla-SPM*, and *bla-SME*, in cattle feces in Bangladesh.

Methods

A cross-sectional study was designed to analyze ARGs in fresh cow dung samples collected from commercial farms and individual houses in four Bangladesh districts, namely, Dhaka, Gazipur, Manikganj, and Tangail. Types of cattle breeds, their existing diseases, recent antimicrobial uses, and vaccine uses were recorded. DNA was extracted from each cow dung sample using commercial kits (Qiagen GmbH, Germany). Real-time quantitative polymerase chain reaction (RT-qPCR) was employed to assess the eight carbapenem resistance genes in the extracted DNA. The eight carbapenem resistance genes in the extracted DNA were assessed by RT-qPCR using the qTOWER3 thermal cycler (Analytik Jena GmbH, Konrad-Zuse-Straße 1, 07745 Jena, Germany).

Results

Group A carbapenemase, *bla-KPC*, was detected in 66.7% of the samples. However, no *bla*-SME was identified in all of the test samples. Group B metallo carbapenemase, *bla-IMP*, *bla-NDM-1*, *bla-VIM*, *bla-SIM*, *bla-GIM*, and *bla-SPM*, were in 66.7% (80/120), 49.2% (59/120), 48.3% (58/120), 68.3% (82/120), 58.3% (70/120), and 12.5% (15/120), respectively. Only 8.3% of the tested samples contained no MBL gene; 10% carried a single-type carbapenemase gene; and the remaining 81.7% carried two or more carbapenemase genes concurrently. Co-carriage of four or more genes was found in over 59% of samples. As many as seven genes were found together in 6.7% of samples. ARG detection in commercial cattle samples and household feces is not statistically significant.

Conclusions

Substantial carbapenem-resistance ARGs were detected in commercially farmed cow dung and household cattle samples. Frequent use of antibiotics for cattle for treatment and prophylactic purposes may influence the high acquisition of ARGs. Bangladeshi cattle farms are reservoirs and routes of AMR, posing a significant threat to the country's public health.

## Introduction

Antimicrobial resistance (AMR) is forecasted to be the leading cause of mortality, morbidity, and financial losses in the upcoming years [1]. Although the emergence of AMR is a natural property, it has been driven by the selection pressure of frequent uses of antimicrobial agents in healthcare, the food chain, agriculture, and fishery [2,3]. Considering the lower cost, availability, and safety, β-lactam antibiotics are currently the most commonly prescribed antimicrobials in humans [4] and cattle farming, particularly to treat bovine mastitis in dairy cow farms [5]. Both humans and animals circumvent the selective toxicity of the β-lactam antibiotics specific for bacterial peptidoglycans and transpeptidases [6]. β-lactam antibiotics belong to natural penicillin and hundreds of other penicillin derivatives such as monobactams, cephalosporins, cephamycins, and carbapenems [7]. Among the currently available β-lactams, carbapenems have become the reliable drug of choice because of their relatively high resistance to hydrolysis by most β-lactamases and robust target capacity for penicillin-binding proteins [8]. The clinically used carbapenems at present are imipenem, meropenem, ertapenem, doripenem, and panipenem, which are used parenterally to treat critical infections by both gram-positive and gram-negative aerobic and many anaerobic bacteria [9]. Reporting of carbapenem resistance has become a tremendous public health concern globally [10]. Several gram-negative bacteria (GNB), such as *Klebsiella pneumoniae*, *Pseudomonas aeruginosa*, and *Acinetobacter baumannii*, are frequently reported to have carbapenem resistance [11], possibly by intrinsic mutations or mediated by transferable carbapenemase-encoding genes [10]. Almost 3,000 different β-lactam-hydrolyzing enzymes are reported [12], commonly called β-lactamases (designated as bla), and function as the primary driving force of resistance in GNB [11,13,14]. The well-known Ambler classification has grouped the β-lactamase enzymes into four molecular classes: A, B, C, and D, based on their primary sequence motif composition [15]. Group A, C, and D β-lactamases use serine at their active site amino acid motifs. At the same time, group B is called metallo-β-lactamases (MBLs) that bind one or two metallic zinc ions essentially at their active sites and aid in breaking down target antibiotics [15,16]. Group A β-lactamases can hydrolyze penicillins, classical cephalosporins, monobactam, imipenem, and meropenems and are further segregated into six subgroups, including *bla-KPC* (*Klebsiella pneumoniae carbapenemase*), *bla-GES* (Guiana extended-spectrum), and *bla-SME* (*Serratia marcescens* enzyme), based on other amino acid composition differences [17]. In contrast, the class B MBL group belongs to the *bla-IMP*, *bla-SPM*, *bla-VIM*, *bla-GIM*, *bla-NDM*, and *bla-SIM* and possesses a broad spectrum for the hydrolysis of almost all β-lactam antibiotics, with the exception of monobactams [18,19]. MBLs were first reported in the 1960s; however, MBL genes in GNB isolated from clinical infections and nosocomial outbreaks drew global attention in 1990 [20]. Since then, there has been increasing reporting of different MBL genes, namely, *bla-IMP*, *bla-VIM*, *bla-NDM*, and *bla-VIM* [21,22]. Reports of MBL gene identification and transmission were focused on clinical bacteria and hospital-associated infections for years [22]. Some recent studies have identified the dissemination of MBL genes from clinical to other environmental spheres [23,24]. Studying the carbapenem resistance gene distribution in the environment is worthwhile for its potential implications for human health and ecological nuisance. Products of cattle farms, such as cow dung and manure, could be classic reservoirs of metagenomic MBL genes or bacteria carrying those ARGs. To our knowledge, limited or no study has been undertaken to evaluate MBL gene prevalence in cow dung samples. This study investigated the prevalence of eight carbapenem resistance genes, *bla-KPC*, *bla-IMP*, *bla-VIM*, *bla-NDM-1*, *bla-SIM*, *bla-GIM*, *bla-SPM*, and *bla-SME*, in fresh cow dung from cattle farms and households in Bangladesh.

## Materials and methods

Study areas and sampling

**Figure 1:**
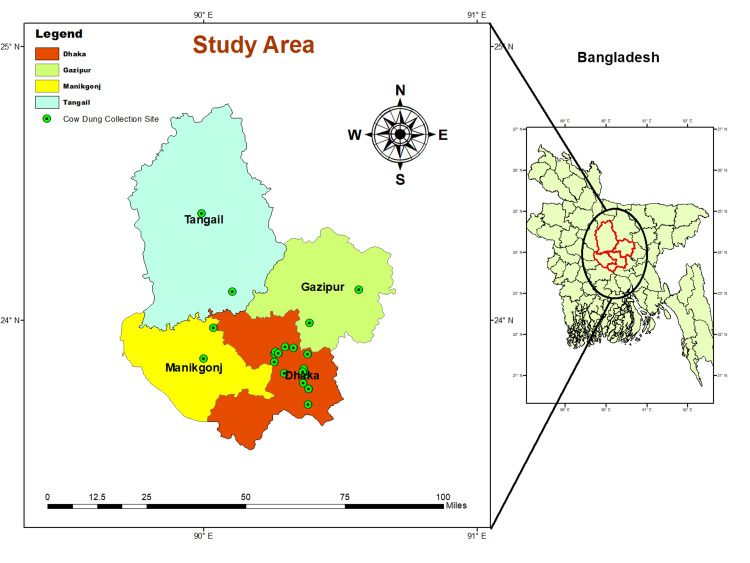
Sampling areas

A cross-sectional study was designed to collect fresh cow dung samples to assess the presence of MBL ARGs from December 2021 to December 2022. One hundred eight cow dung samples were collected from 20 commercial cattle farms. Twelve cow dung samples were collected from six individual houses. The history of current diseases and medicine usage was recorded using a brief, structured questionnaire. The sampling sites were chosen from some dairy farming areas located in four districts in Bangladesh, namely, Dhaka, Gazipur, Manikgang, and Tangail (Figure 1). Following all safety precautions and aseptic techniques, cow dung samples were collected to avoid probable cross-contamination. Samples were taken in clean, pre-labeled stool specimen containers, stored immediately in insulated ice boxes, and transported to the One Health Laboratory of the Department of Microbiology, Jahangirnagar University, Savar, where subsequent molecular biology analyses were carried out.

Geographic locations of cattle farms (n=20) and houses (n=6) were shown from where cow dung samples were collected. Sampling sites include four districts in Bangladesh and are displayed on the map. Geographic information mapping software, ArcGIS version 10 for Windows (Esri, CA, USA), was used to draw the sampling site-location map.

DNA extraction from cow dung samples

Approximately one gram of each cow dung was suspended in 3 mL of sterile phosphate-buffered saline using a sterile spatula and mixed well. The QIAamp DNA stool mini kit (Qiagen GmbH, Germany) was used to manually extract DNA from resuspended cattle feces following the manufacturer's protocol. The quick purification of nucleic acids is made possible by the practical QIAamp spin-column method. The extracted DNA was eluted in 200 µl of elution buffer and preserved at -20°C for next-level analyses. Separate aliquots of extracted DNA were stored in a repository at -80°C for any future research.

Primer design

Eight pairs of MBL primer sequences (*bla-KPC*, *bla-IMP*, *bla-VIM*, *bla-NDM-1*, *bla-SIM*, *bla-GIM*, *bla-SPM*, *bla-SME*), their annealing temperatures, and amplicon lengths were obtained from previous literature [25]. The conformities of the oligonucleotide primer sequences were further checked in the NCBI BLAST (Basic Local Alignment Search Tool) database and then synthesized from an external manufacturer (Macrogen Inc., South Korea).

Real-time polymerase chain reaction amplification program

Real-time quantitative polymerase chain reaction (RT-qPCR) was used to determine relative (per 16S rRNA gene) abundances of ARGs in DNA extracted from cow dung samples. The qTOWER3 thermal cycler (Analytik Jena, GmbH, Germany) was used to amplify the qPCR. For each qPCR reaction, extracted DNA 1.0 µl was added to 10 µl of Go Taq qPCR master mix (Promega Corporation, 2800 Woods Hollow Rd, Fitchburg, WI 53711, United States), and five µmol of each primer (2 µL), and the nuclease-free water was added to make a final volume of 20 µL. The optimal program of the qPCR includes an initial denaturation at 95°C for two minutes, followed by 45 cycles of denaturation at 95°C for 15 seconds; an annealing temperature of 58°C was used to amplify genes *bla-KPC*, *bla-IMP,* *bla-VIM,* and *bla-NDM-1 *and 56°C for the genes *bla-SIM, bla-GIM, bla-SPM*, and *bla-SME* for 15 seconds and an extension at 60°C for 20 seconds. The amplification process was completed with a melting step that was carried out using the following cycling parameters: 60°C for 15 seconds and 5°C temperature adjustments up to a final temperature of 95°C. SYBR green's fluorescence energy was used to measure the amount of amplified product. SYBR green binds double-stranded DNA after extension by PCR and emits fluorescence. A negative control (no template control) was used in each PCR run. The bacterial 16S rRNA gene was used as a positive control.

Determination of specificity and sensitivity of the qPCR

The following equations were used to calculate specificity and sensitivity: specificity = [D/(C + D)]*100 and sensitivity = [A/(A + B)]*100, where A is a true positive, B is a false negative, C is a false positive, and D is a true negative (Table 1). The standard curve approach obtained the correlation coefficients' R2 values. All the genes had R2 values of 0.98.

**Table 1:** Specificity and sensitivity of the qPCR detection system for carbapenemase genes %, percentage; A, true positive; B, false negative; C, false positive, D, true negative. The sensitivity and specificity of each carbapenem gene detection were determined based on the negative control results. No template was added for real-time quantitative polymerase chain reaction (RT-qPCR) amplification and negative control results, where known bacterial 16S rRNA gene was used with respective primer pairs

ARG analyzed	True (+) = A	False (-) = B	False (+) = C	True (-) = D	% sensitivity; specificity*
bla-KPC	80	0	0	40	100; 100
bla-IMP	80	0	0	40	100; 100
bla-NDM-1	59	0	0	61	100; 100
bla-VIM	58	0	0	62	100; 100
bla-SME	0	0	0	120	100; 100
bla-SPM	15	0	0	105	100; 100
bla-GIM	70	0	0	50	100; 100
bla-SIM	82	0	0	38	100; 100

Statistical analysis

Descriptive and inferential statistics determined the MBL genes in different cow dung samples. Descriptive statistics were reported as frequencies and percentages. Pearson's chi-square test was used to test the association between ARG carriage in farm-based cow dung and household cow dung. A two-tailed p-value of 0.05 or smaller was considered statistically significant. SPSS Statistics version 20.0 (IBM SPSS Statistics for Windows, Version 20.0. Armonk, NY: IBM Corp.) was used to analyze all the data.

Ethics statement

This study was an extension of a similar previous research project approved by the Ethics and Research Review Committee of the Jahangirnagar University Faculty of Biological Sciences (approval number: BBEC, JU/M 2017 12(4), approval date: December 27, 2017). The ethical committee has waived the requirement for new approval. The study followed all the ethical guidelines and regulations for environmental samples. Informed verbal consent was obtained from each cattle owner/farm manager for collecting the cow dung samples and information from their farms/cattle. Farm identities were kept anonymous to protect commercial, personal, and private information. A sample identification code was assigned correctly for each sample collected.

## Results

Study farms and samples

Out of 120 samples, 108 (90%) cow dung samples were collected from 20 commercial cattle farms. The farms were rearing a mixture of dairy and beef-producing cattle, namely, Holstein Friesian, Holstein Friesian Jersey, Sahiwal, and their breeds. Some farms raise well-known indigenous breeds like Red Chittagong cattle, Munshiganj cattle, and Pabna cattle. Twelve cow dung samples (10%, 12/120) were collected from individual houses raising non-described local cattle. Two cattle were foot-rot-infected, and seven had fevers during the sample collection. All except four cattle had antibiotic exposure within the last six months. The cattle were being treated with homeopathy for years. Antibiotic use history was almost similar between the cattle of commercial farms and backyard farms. Over 96% (116/120) of the cattle were vaccinated for either anthrax, foot and mouth diseases, or black quarter.

Distribution of carbapenemase genes in cow dung samples

This study measured the presence of eight critical carbapenemase genes in 120 cow dung samples collected from 20 Bangladeshi cattle farms and six individual houses. We found only 10 cow samples where no MBL gene was detected; the remaining 110 samples were detected with at least one MBL gene out of eight genes investigated. Group A carbapenemase, *bla-KPC*, was detected in 66.7% of the samples. However, *bla-SME* was identified in all of the test samples. In contrast, Group B metallo carbapenemases, *bla-IMP*, *bla-NDM-1*, *bla-VIM*, *bla-SIM*, *bla-GIM*, and *bla-SPM*, were in 66.7% (80/120), 49.2% (59/120), 48.3% (58/120), 68.3% (82/120), 58.3% (70/120), and 12.5% (15/120), respectively (Table 1). The newly optimized qPCR technique identified all the carbapenemase genes in the test samples. We calculated the sensitivity and specificity of each carbapenem gene detection by RT-qPCR cycle. Deionized water was used as the negative control template, and a known bacterial 16S rRNA gene was used with respective primer pairs as the positive control. A cut-off cycle threshold (Ct) value of 30 was determined to be selected as ARG-detection positive. No expected deviation was found in amplification cycles for positive and negative controls. Hence, the qPCR's specificity and sensitivity were 100% (Table 1).

**Figure 2:**
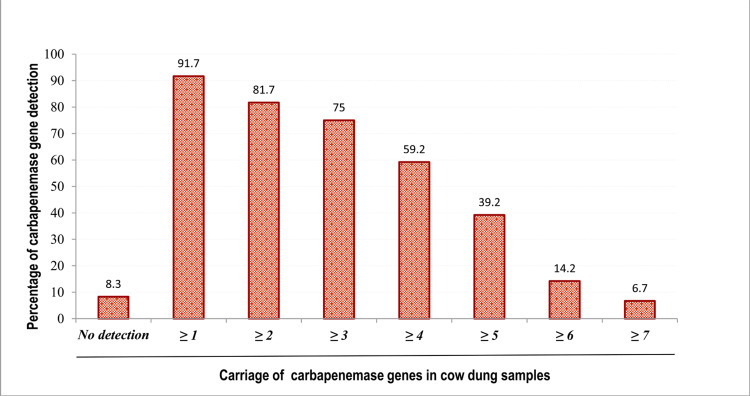
Cumulative distribution of carbapenemase genes in cow dung samples The value bar shows the percentage prevalence. Only 8.3% where no metallo-β-lactamase (MBL) gene was detected, 10% of the tested samples were found to carry single type carbapenemase gene, and the remaining 81.7% of samples were carrying two or more carbapenemase genes concurrently. Co-carriage of four or more genes was found in over 59% of samples. As many as seven genes were found together in 6.7% of samples

Only 10% of the tested samples carried single-type carbapenemase genes; the remaining 81.7% carried two or more carbapenemase genes concurrently. Co-carriage of four or more genes was found in over 59% of samples. As many as seven genes were found in 6.7% of samples (Figure 2).

Comparative distribution of carbapenemase genes in farm-based and household cow dungs

**Figure 3:**
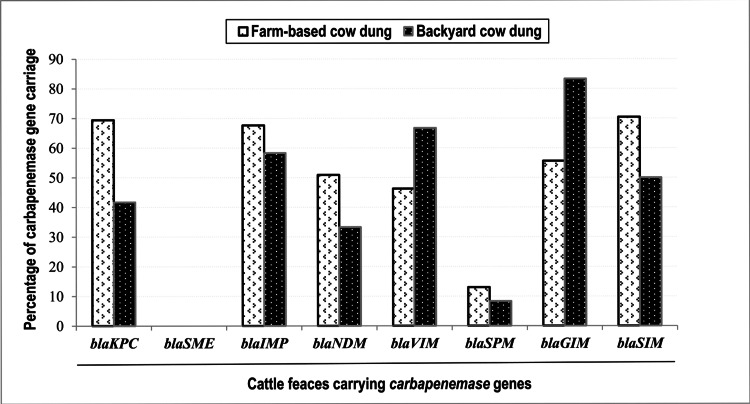
Comparative distribution of carbapenemase genes in farm-based and household cow dung samples Five carbapenem resistance genes, *bla-KPC, bla-IMP, bla-NDM, bla-SPM*, and *bla-SIM*, were found to be higher in commercial samples. The two carbapenem resistance genes, *bla-VIM *and *bla-GIM*, were exhibited higher in household cow dung samples. The differences in antimicrobial resistance gene (ARG) prevalences are not statistically significant. One Group A carbapenem resistance gene, *bla-SME*, was neither identified in commercial cow dung samples nor household cow dung samples

From the assessment of 108 cow dung samples from commercial farms, the prevalence of *bla-KPC*, *bla-IMP*, *bla-NDM*, *bla-VIM*, *bla-SPM*, *bla-GIM*, and *bla-SIM* was detected in 69.4%, 67.6%, 50.9%, 46.3%, 13%, 55.6%, and 70.4%, respectively. No significantly different prevalences of the genes were noticed in household cow dung samples. Five genes, *bla-KPC*, *bla-IMP*, *bla-NDM*, *bla-SPM*, and *bla-SIM*, were higher in commercial samples, and the other two genes exhibited the reverse prevalence (Figure 3).

## Discussion

We investigated the contamination of AMR traits, focusing on eight carbapenemase genes, in both commercial cattle farm-based cow dung and home-based backyard farming cattle cow dung collected from four districts of Bangladesh. The study showed that over 90% of the tested cow dung samples carried some hazardous carbapenemase gene. Carriage of both serine-based Grou A and zinc metal-based Group B carbapenemase (MBL) genes was there. The prevalence of the ARGs in the cow dung samples varied substantially, with no detection for *bla-SME* and low detection for *bla-SPM*. The *bla-SME* and *bla-SPM* were reported as less common minor carbapenemase genes along, with *bla-GIM* and *bla-SIM* [26,27]. Unexpectedly, our study has identified a high prevalence of *bla-GIM* and *bla-SIM* together with the classical carbapenemase genes *bla-IMP*, *bla-NDM*, and *bla-VIM* [22,28].

More than half of the samples had four or more carbapenemase genes together. Some earlier studies reported some ARGs of β-lactam resistance, quinolone resistance, and colistin resistance in organic manures [29]; however, reporting the carbapenem resistance gene at this high abundance has not been reported yet. The abundance of carbapenem-resistance genes in Bangladeshi animal husbandry substantiates earlier studies stating that Indian subcontinental countries are endemic with carbapenemase genes in the human-animal-environment interface [22,30]. The MBL-encoding genes are generally transmitted by mobile genetic elements, facilitating rapid horizontal gene transfer (HGT) among different bacteria [28]. Therefore, our identified MBL genes may be disseminated from the cow dung reservoir to other domains of One Health, which link the health of animals, humans, plants, and environments in an interdependent cycle [31]. The high concentration of AMR hazards can easily be exposed to humans via direct or indirect contact with the climate, soil, agriculture, or food and water cycles. When these ARGs containing cow dung are disposed of in water or a nearby environment or used for agriculture/garden manure, there is a high possibility of spreading the ARGs to other pathogens through vertical or HGT. This study has set a surveillance framework for ARG screening tests in microbiology culture-free settings by rapid RT-qPCR techniques. The study framework is considered a step toward the sustainable development goal of surveillance of AMR under the One Health approach for developing countries, as emphasized by the tripartite alliance of the World Health Organization, the Food and Agriculture Organization, and the World Organization for Animal Health [32].

When adopting this study, we hypothesized that commercial farm-origin cow dung would be a reservoir of higher carbapenem resistance genes than household-origin cow dung. Our study did not find significant differences in carbapenem resistance genes in terms of abundance and diversity in the two samples. Our study has identified that the uses of antibiotics for cattle in commercial farms and individual houses are almost similar. Cephalosporin-group antibiotics were found to be the drug of choice for both cattle farms and household settings. As all the antibiotics are available over the counter in Bangladesh, people frequently buy and consume antibiotics in their jurisdiction. Used antibiotic sachets and parts of unused antibiotics are commonly disposed of in nearby household garbage. These discrepantly exposed antibiotics may contribute to the high acquisition of ARGs in food-producing animals and the surrounding environments [33,34]. As reported, reducing antimicrobial usage often results in diminished AMR emergence [35], and some policies are imperative to reduce the AMR burden in cow dung.

Compared to traditional phenotypic and conventional approaches, a qPCR system provides a quicker and more effective technology for determining antibiotic susceptibility. We looked into the possibility of identifying significant genes for antibiotic resistance in cow dung samples without culture. Our qPCR system has the advantage of detecting the target ARGs with 100% sensitivity and 100% specificity, as affirmed by earlier studies [36,37].

Limitations of the study

This study had a few common limitations. The study was conducted under a cross-sectional study design, and a follow-up assessment was not carried out due to resource limitations. This study did not assess the acquisition of ARGs in bacterial communities by microbiological investigation. Future research will be crucial to determining the harborage of ARGs in bacteria and their associated phenotypic antimicrobial susceptibilities. This study analyzed the distribution of eight carbapenem-resistance genes, leaving behind a few unrelated gene variants that were not investigated. The small sample size remains an inherent constraint to conducting well-strength statistical analyses. However, our studies generated data by maintaining internal validity by repeating independent experiments when necessary. We believe the findings shed some light on the potential source of disseminating critical carbapenem resistance genes at the animal-human interface.

## Conclusions

To the best of our knowledge, our study successfully implemented the first qPCR assays to identify carbapenem resistance genes in Bangladeshi food at animal-human interfaces. Thus, our current efforts have laid the foundation for surveillance of ARG detection at the animal-human interface; the approach should be continued with comprehensive coverage. Our study provides the groundwork for doable, simple, and affordable qPCR-based surveillance of AMR that could be conveniently implemented in low- and middle-income countries. The study design can be further expanded for other One Health domains. To preserve the efficacy of carbapenems as last-resort antibiotics, further detailed AMR surveillance and associated national policy are urgently required to focus on carbapenem resistance.
